# A bibliometric analysis of cardiomyocyte apoptosis from 2014 to 2023: A review

**DOI:** 10.1097/MD.0000000000035958

**Published:** 2023-11-24

**Authors:** Rui Wang, Xu Luo, Songyun Li, Xin Wen, Xin Zhang, Yunxiang Zhou, Wen Xie

**Affiliations:** a Chengdu University of Traditional Chinese Medicine, Chengdu, China; b Hospital of Chengdu University of Traditional Chinese Medicine, Chengdu, China.

**Keywords:** bibliometric, cardiomyocyte apoptosis, CiteSpace, visualization, VOSviewer

## Abstract

Cardiomyocyte apoptosis is an important factor in cardiac function decline observed in various cardiovascular diseases. To understand the progress in the field of cardiomyocyte apoptosis research, this paper uses bibliometrics to statistically analyze publications in this field. A total of 5939 articles were retrieved from the core Web of Science database, and then VOSviewer and Citespace were used to conduct a scientometric analysis of the authors, countries, institutions, references and keywords included in the articles to determine the cooperative relationships between researchers that study cardiomyocyte apoptosis. At present, the research hotspots in this field mainly include experimental research, molecular mechanisms, pathophysiology and cardiac regeneration of cardiomyocyte apoptosis-related diseases. NOD-like receptor thermal protein domain associated protein 3 inflammasome, circular RNA, and sepsis are the research frontiers in this field and are emerging as new areas of research focus. This work provides insight into research directions and the clinical application value for the continued advancement of cardiomyocyte apoptosis research.

## 1. Introduction

Cardiomyocytes are the main functional cells of cardiac contraction and the key to maintaining the normal pumping function of the heart. In mammals, precipitated cardiomyocytes are considered terminally differentiated cells with very little ability to repair or undergo division. Once the myocardium is damaged, it is difficult to recover.^[1,2]^ A special type of programmed cell death, such as apoptosis, pyroptosis, autophagy, programmed necrosis, and ferroptosis, called myocardial apoptosis is common in cardiomyocytes and is crucial in cardiac development.^[3,4]^ Myocardial damage caused by abnormal myocardial apoptosis is one of the primary factors that aggravates cardiovascular disease. The stimulation of hypoxia,^[5,6]^ inflammatory factors,^[7,8]^ angiotensin II,^[9,10]^ and other factors induce excessive myocardial apoptosis, irreversible myocardial structural changes, damage myocardial contraction characteristics, and even cause heart failure, atherosclerosis, ischemia–reperfusion (I/R) and a series of lesions.^[11–13]^ Therefore, it is of great significance to clarify the mechanism of cardiac cell death and determine the intervention methods to reduce myocardial cell apoptosis and prevent myocardial disease.

In this study, CiteSpace and VOSviewer were used to collect information on myocardial apoptosis and draw knowledge maps. The 2 software programs have complementary advantages. CiteSpace is a bibliometric software program based on the JAVA application. It combines medical informatics, data mining and information visualization technology and uses interactive tools to extract citation data. By constructing a progressive knowledge domain map, it intuitively shows the research hotspots and hidden knowledge within scientific literature and helps researchers quickly and accurately predict research prospects and emerging trends.^[14,15]^ VOSviewer is also one of the most commonly used visualization tools.^[16]^ It can be used to produce maps of countries, authors, journals, keywords, and references. It has the characteristics of simple operation and producing beautiful images.

Bibliometrics is an important approach that uses statistical methods to quantitatively or semiquantitatively analyze the literature to reveal the distribution of literature and predict the direction of discipline development.^[17,18]^ This study intends to summarize and refine the relevant scientific and technological literature on myocardial apoptosis in the Web of Science database from the perspective of bibliometrics, describe the existing achievements of international research on myocardial apoptosis, and reveal the current research status of myocardial apoptosis from a macro perspective. Moreover, this work is intended to be used as a reference for future related research.

## 2. Methods

### 2.1. Data collection

**Figure 1.**
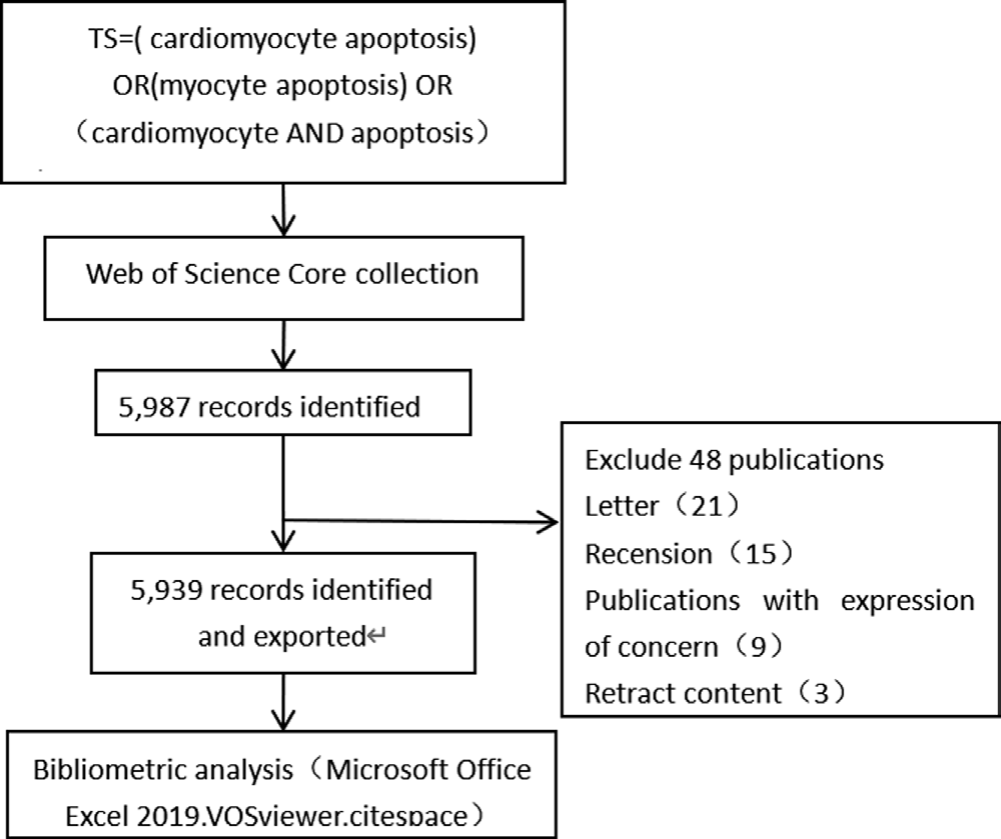
Flowchart illustrating the search strategy and selection process in cardiomyocyte apoptosis.

The Web of Science is an internationally recognized high-quality database of reaction science research standards that is recognized by many scholars and is suitable for data analysis and research.^[19]^ Therefore, the data of this paper are derived from the Science Citation Index Expanded and Social Sciences Citation Index in the core collection citation index of the Web of Science. The search term was = “Cardiomyocyte apoptosis ‘OR’ Myocyte apoptosis ‘OR’ (cardiomyocyte ‘AND’ apoptosis).” To avoid deviations caused by daily data updates, the search time was set from January 1, 2014 to June 1, 2023. A total of 5987 articles were obtained, and 5939 articles were finally used in the study. The articles included 8 different types of literature. There were 5264 research papers, accounting for 88.6% of the total. Reviews were the second most common type (n = 551), accounting for 9.3% of the total record. The remaining 6 types of literature included conference abstracts (n = 103), online publications (n = 60), editorial materials (n = 19), conference proceedings papers (n = 16), book chapters (n = 14), and retrieved publications (n = 29). The 5939 documents were exported in full, including the cited references, in download _.txt format and saved to a folder. The specific flow chart describing the approach is shown in Figure 1.

The 5939 papers used in this study are from 85 countries, published in 920 journals, written by 28,592 authors from 4129 institutions, and cite 171,712 papers from 8949 journals.

## 3. Results

### 3.1. Temporal distribution map of the literature

**Figure 2.**
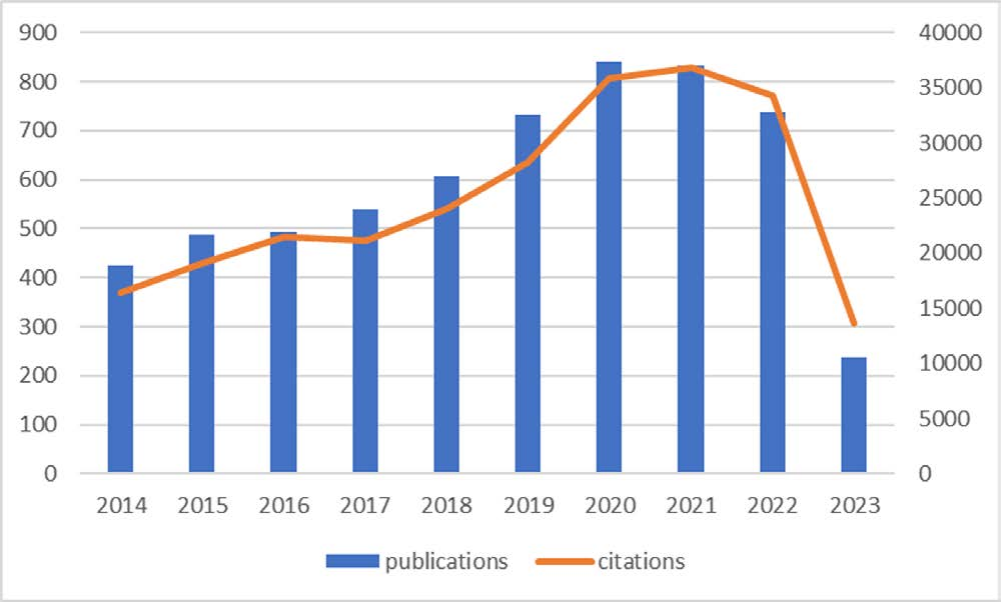
Trends in the growth of publications in cardiomyocyte apoptosis.

The number of studies published in a certain time interval can reflect the research status and development trend of the field. This article selected the literature published from 2014 to 2023 for analysis. As shown in Figure 2, the largest number of publications was in 2020, with a total of 842 articles. Because the date of inclusion is limited to June 1, the number of publications in 2023 is the lowest, at 238, while the average annual number of publications is 593.90. Figure 2 shows that the overall number of publications in this field is increasing, and was especially high in 2020 and 2021, indicating that the research interest in this field is not decreasing. Additionally, the total number of citations varies with the number of publications.

### 3.2. Distribution of countries/regions

As shown in Table 1, the country with the largest number of publications is China, followed by the United States and Canada, accounting for 42.99%, 31.65%, and 5.99% of the total publications, respectively. These 3 countries account for nearly four-fifths of the total number of publications, indicating that they have made great contributions to research in this field. According to the average literature citation results, the United Kingdom (47.19), the Netherlands (46.56), and Germany (44.60) are the 3 countries with the highest average citations, indicating that the quality of research emerging from these 3 countries is high. Among the top 10 countries, there are 5 countries with a connection degree greater than 0.1, namely, the United States (0.40), the United Kingdom (0.17), Germany (0.13), France (0.12), and China (0.12), indicating that these 4 countries have very important positions in this field of research.

**Table 1:** Top 10 most productive countries/regions in cardiomyocyte apoptosis.

Rank	Countries/regions	Documents	Citations	Average citation/publication	Total link strength	Centrality
1	China	4151	77,221	18.6	689	0.1
2	USA	1061	37,450	35.3	804	0.4
3	Canada	175	5045	28.83	139	0.13
4	Germany	174	5431	31.21	215	0.17
5	United Kingdom	157	3815	24.3	229	0.04
6	Italy	144	5810	40.38	173	0.02
7	Japan	144	3638	25.26	104	0.01
8	India	134	2251	16.8	123	0.12
9	South Korea	83	1369	16.5	49	0.06
10	Iran	82	1431	17.45	43	0.03

**Figure 3.**
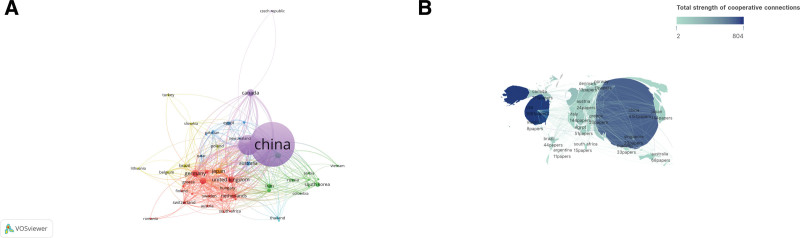
Cooperation map of countries/regions in cardiomyocyte apoptosis. (A) A visual map for the VOSviewer network. (B) A visual map for Scimago Graphica.

The VOSviewer control panel setting parameters used were as follows: analysis type was cooperative coauthorship, analysis unit was country, minimum number of national documents was 5, other is the default value. Data were retrieved from 85 countries, of which 51 meet the threshold, and were used to generate a network visualization map, as shown in Figure 3A. Each node represents a country, the size of the node represents the number of documents published by researchers from the country, the color represents a cluster, the nodes are connected by curves, and the thickness of the line represents the number of connections between the nodes. Figure 3A shows that China mainly cooperates closely with the United States, Canada, Britain, India, Japan and South Korea; The United States mainly cooperates closely with China, Italy, Canada, Germany, Britain, Australia, France, and Japan; Canada mainly cooperates closely with the United States, China, Greece, Brazil, France and Saudi Arabia. Scimago Graphica was used to make the national cooperative geographic visualization map, and Marks was set to the field map. The total strength of the national cooperative connection was set to green and blue. The weaker the connection strength is, the closer it is to green, the stronger it is, and the closer it is to blue. As shown in Figure 3B, the intensity of U.S. cooperation ranks first, followed by China.

### 3.3. Distribution of authors and research institutions

As shown in Table 2, Huang, Chih-yang (62) and Kuo, Wei-wen (44) from China Medical University ranked first and third in terms of the number of published papers, and Ren, Jun (50) from the General Hospital of the People’s Liberation Army District ranked second. However, in terms of the average number of citations, Wang, Kun (78.61) from Qingdao University and Zhou, Hao from the General Hospital of the People’s Liberation Army District were the 2 top ranked authors.

**Table 2:** Top 5 authors based on publications (Rank_a_) and citations (Rank_b_).

Rank_b_	Author	Documents	Citations	Average citation/publication	Total link strength	Centrality	H-index
1_a_	Huang, Dhih-Yang	62	1255	20.24	146	0	46
2_a_	Ren, Jun	50	1821	36.42	35	0.09	84
3_a_	Kuo, Wei-wen	44	1042	23.68	117	0	37
4_a_	Gao, Erhe	42	2002	47.67	57	0.14	54
5_a_	Li, Yan	38	788	20.74	29	0.06	32
1_b_	Wang, Kun	26	2044	78.61	25	0.04	29
2_b_	Zhou, Hao	18	1338	74.33	14	0.02	45
3_b_	Li, Na	20	1438	71.9	28	0.09	19
4_b_	Deng, Wei	16	987	61.69	26	0.03	5
5_b_	Wang, Hao	22	1282	58.27	10	0.01	14

**Figure 4.**
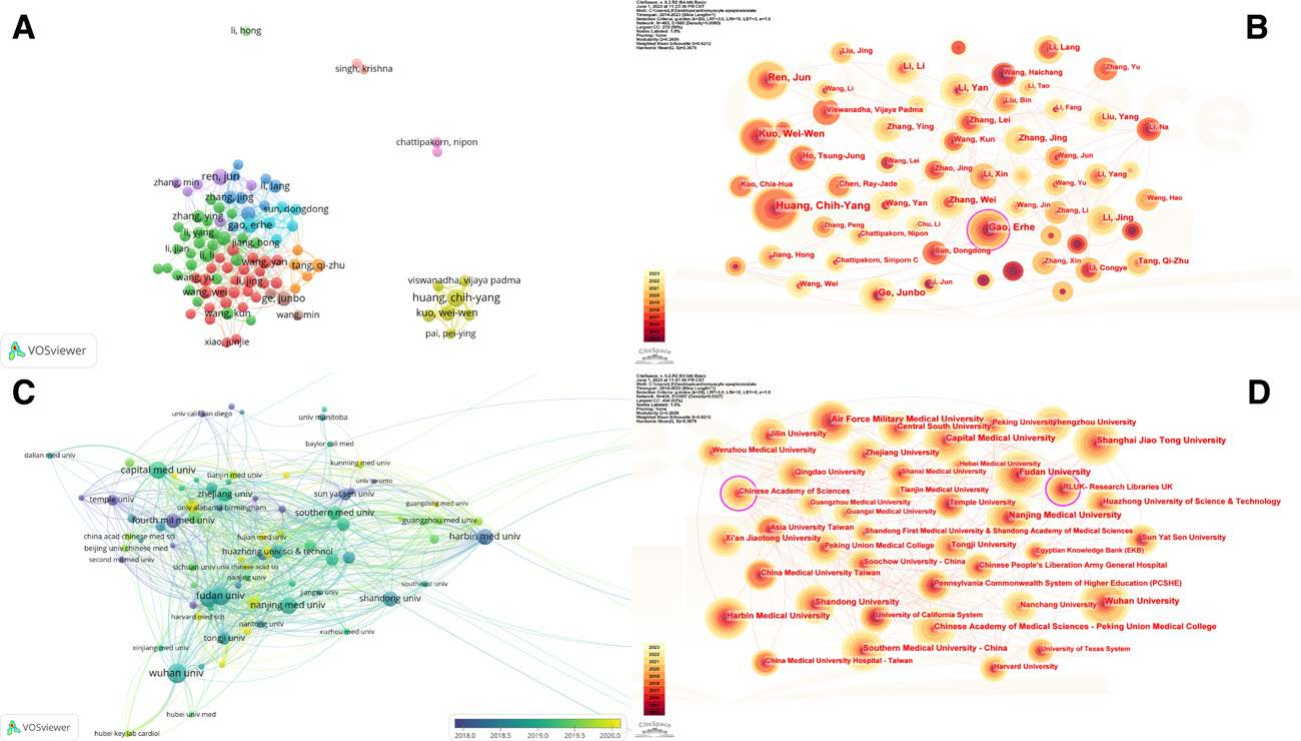
Collaboration networks among authors and institutions researching cardiomyocyte apoptosis. (A) A visual map for the VOSviewer network among authors. (B) A visual map for the CiteSpace network among authors. (C) A visual map for the VOSviewer network among institutions. (D) A visual map for the CiteSpace network among institutions.

The VOSviewer control panel parameter settings used were: the author’s minimum number of documents. A total of 1,528,592 authors were searched, and 95 authors reached the threshold standard. Based on the cooperative analysis, the authors were divided into different clusters and colors according to the time of the author’s appearance, and the time was superimposed for visual analysis. As shown in Figure 4A, different nodes represent different authors. The larger the node is, the greater the number of articles published by the author. The connection curve between the nodes represents the cooperative relationship between authors. The thicker the curve, the stronger the cooperative relationship. Huang, Chih-yang cooperated closely with Kuo, Wei-wen, Ho, Tsung-jung, Chen, Ray-jade, Kuo, and Chia-hua. Ren, Jun worked closely with Zhou, Hao, Wang, Jin, Yang, Jian, Zhang, Yingmei, Liu, Yang, etc.; Kuo, Wei-wen and Huang, Chih-yang, Viawanadha, Vijaya padma, Chen, Ray-jade, Ho, Tsung-jung and other authors with close cooperation; Wang, Kun and Li, Peifeng, Wang, Tao, Zhang, Jian, Liu, Jing, and Li, Qian were observed to cooperate closely; Zhou, Hao worked closely with Wang, Hao, Zhang, Li, Li, Lei, Zhang, Peng, and Zhang, Jun. The CiteSpace parameters were set as follows: time slicing (2014–2023), year per slice (1), term source was all, g-index (20), Top-N (50), and all other parameters were set to default values. Figure 4B is the author’s network visualization map. The size of the node represents the number of posts, and different colors represent different years. From 2014 to 2023, the color changes from red to yellow, and Spotlight is clicked. A node with betweenness centrality ≥ 0.1 was found to be Gao, Erhe from Temple University, indicating that this author played an important role in the field of cardiomyocyte apoptosis.

As shown in Table 3, Wuhan University has the largest number of papers, with 188 papers, followed by Fudan University (172) and Capital Medical University (156). Assessment of the average number of citations, the “University of California, San Diego” to have the highest citations, with 34 papers averaging 88.62 citations, with the “Chinese Academy of Sciences” ranked second, with 50 papers averaging 53.72 citations.

**Table 3:** Top 5 institutions based on publications (Rank_a_) and citations (Rank_b_).

Rank	Institutions	Documents	Citations	Average citation/publication	Total link strength
1_a_	Wuhan University	188	3889	20.69	110
2_a_	Fudan University	172	5054	29.38	182
3_a_	Capital Medical University	156	2327	14.92	114
4_a_	Shanghai jiao tong University	155	4307	27.79	120
5_a_	Nanjing Medical University	149	3156	21.19	123
1_b_	University of California, San Diego	34	3013	88.62	12
2_b_	Chinese Academy of Sciences	50	2686	53.72	74
3_b_	University of Minnesota	21	1079	51.38	25
4_b_	University of Chinese Academy of Science	22	1109	50.41	40
5_b_	Guilin Medical University	22	1062	48.27	22

The VOSviewer software settings were as follows: the minimum number of institutions was 20. After screening 4129 institutions, the number of institutions that met the threshold was 94, as shown in Figure 4C. The institutions within each group worked closely together to form a total of 8 cooperative groups. The first group consisted of Wuhan University, Hubei Key Laboratory of Cardiology, Hubei Medical University, Xinjiang Medical University, Chongqing Medical University, Nanjing University of Chinese Medicine, etc. The second group comprised Shanghai Jiao Tong University, Fourth Military Medical University, Chinese People’s Liberation Army University, Temple University, Thomas Jefferson University and Third Military Medical University. The third group comprised Capital Medical University, Hebei Medical University, Dalian Medical University, Zhengzhou University, Peking University and Xian jiao tong University. The fourth group comprised Fudan University, Tongji University, Nantong University, Harvard Medical School, University of Wyoming, Shanghai University and so on. The fifth group consisted of Zhejiang University, Tianjin Medical University, the University of Cincinnati, University of California, San Diego, Dzhk German Cardiovascular Research Center, Kings College of London, etc. The sixth group comprised Huazhong University of Science and Technology, Central South University, Southern Medical University, Sun Yat-sen University, University of Toronto and University of South China. The seventh group comprised Harbin Medical University, Jilin University and Jinzhou Medical University. The eighth group comprised China Medical University, National Taiwan University, Asia University, China Medical University Hospital, Taipei Medical University and so on. Additionally, a lack of cooperation between countries was observed. To investigate this, the CiteSpace parameters were set as follows: time slicing (2014–2023), year per slice (1), term source is all, and other parameters were set to default values. Figure 4D shows the visual map of the results. It can be clearly seen that the nodes of the purple outer ring, including the Chinese Academy of Sciences and RLUK-Research Libraries UK, are nodes with betweenness centrality > 0.1. This means that these 2 institutions are also important institutions for the study of cardiomyocyte death.

### 3.4. Cocited references and reference bursts

**Figure 5.**
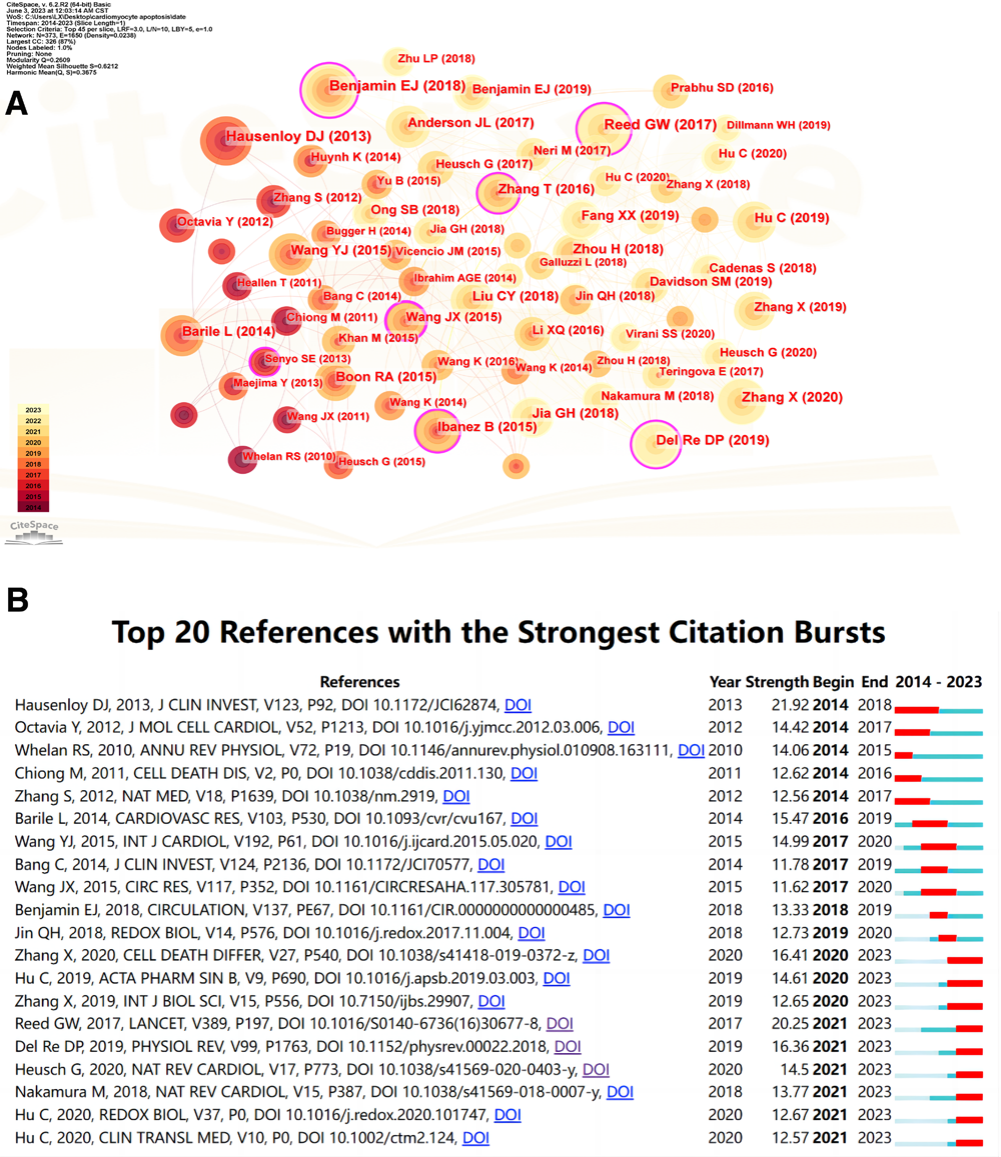
(A) Reference cocitation network in cardiomyocyte apoptosis. (B) Top 20 references with the strongest citation bursts in cardiomyocyte apoptosis.

The CiteSpace parameters were set as follows: time slicing (2014–2023), year per slice (1), term source is all, and selection criteria (TopN = 45). Using to the above settings, a network map with N = 373 and E = 1650 (density: 0.0238) was obtained. As shown in Figure 5A, the size of the node is related to the citation frequency. The node with a purple outer ring has a high degree of betweenness centrality, which represents the size of its influence to a certain extent. The literature that determines the 2 classifications based on the most frequently cited and the size of the betweenness centrality is shown in Table 4. The most cited publication is “the Heart Disease and Stroke Statistics-2017 Update: A Report From the American Heart Association” (90)^[20]^ published in Circulation, with a betweenness of 0.12, followed by “Acute myocardial infarction” (81)^[21]^ published in the Lancet, with a degree of betweenness of 0.07. The literature with the highest betweenness centrality was published in Circulation Research “MicroRNA-103/107 Regulate Programmed Necrosis and Myocardial Ischemia/Reperfusion Injury Through Targeting FADD” (0.21).^[22]^ This was followed by a publication in Nature Medicine entitled “CaMKII is a RIP3 substrate mediating ischemia-and oxidative stress-induced myocardial necroptosis” (0.17).^[23]^ Figure 5B shows the top 20 publications with the strongest citation bursts, with the Lancet’s “Acute myocardial infarction article” having the highest citation strength of 20.25.^[21]^

**Table 4:** Ranking of the top 5 cited references of publications based on frequency (Rank_a_) and centrality (Rank_b_).

Rank	Frequency	Centrality	Title	Journal	Author	Year
1_a_	90	0.12	Heart disease and stroke statistics-2017 update: a report from the American Heart Association	CIRCULATION (IF: 39.918)	Benjamin EJ	2018
2_a_	81	0.11	Acute myocardial infarction	LANCET (IF: 202.731)	Reed GW	2017
3_a_	72	0.07	FNDC5 alleviates oxidative stress and cardiomyocyte apoptosis in doxorubicin-induced cardiotoxicity by activating AKT	CELL DEATH DIFFER (IF: 12.061)	Zhang X	2020
4_a_	68	0.12	Fundamental mechanisms of regulated cell death and implications for heart disease	PHYSIOL REV (IF: 46.500)	Del Re DP	2019
5_a_	59	0.01	Acute myocardial infarction	NEW ENGL J MED (IF: 202.731)	Anderson JL	2017
1_b_	42	0.21	MicroRNA-103/107 regulate programmed necrosis and myocardial ischemia/reperfusion injury through targeting FADD	CIRC RES (IF: 23.213)	Wang JX	2015
2_b_	50	0.17	CaMKII is a RIP3 substrate mediating ischemia- and oxidative stress-induced myocardial necroptosis	NAT MED (IF: 87.241)	Zhang T	2016
3_b_	55	0.15	Evolving therapies for myocardial ischemia/reperfusion injury	J AM COLL CARDIOL (IF: 27.203)	Ibanez B	2015
4_b_	90	0.12	Heart disease and stroke statistics-2017 update: a report from the American heart association	CIRCULATION (IF: 39.918)	Benjamin EJ	2018
5_b_	68	0.12	Fundamental mechanisms of regulated cell death and implications for heart disease	PHYSIOL REV (IF: 46.500)	Del Re DP	2019

Usually, the cited literature represents the knowledge base of the field, so the highly cocited articles are representative of the knowledge base of cardiomyocyte apoptosis. “Heart Disease and Stroke Statistics-2017 Update: A Report From the American Heart Association”^[20]^ was the most cited article. The American Heart Association (AHA) is one of the most important societies in the field of cardiology and stroke. The AHA is committed to the prevention and treatment of heart disease and stroke and provides relevant guidelines that are updated quickly. The content published by the AHA incudes information about health factors, disease risk factors, and important heart and circulatory system diseases as well as updated data (including medium score, congenital heart disease, arrhythmia, coronary heart disease, heart failure, heart valve disease, etc.) and related research results.

The article “Acute myocardial infarction”^[21]^ is a review written by Eric Boersma, Nestor Mercado and others and published in the Lancet. It focuses on the current epidemiology, risk factors and population management of acute myocardial infarction and provides a medical reference for the long-term survival of such patients.

“MicroRNA-103/107 Regulate Programmed Necrosis and Myocardial Ischemia/Reperfusion Injury Through Targeting FADD”^[22]^ is also a basic research article written by Wang et al that describes that necroptosis is the most common form of programmed cell death in heart failure and I/R. Fas-associating protein with a novel death domain is involved in the formation of death-induced signal complexes and binds to the death domain to induce exogenous cell death. MiRNAs and long non-coding RNAs play an important role in the biological processes of cardiac cells and may regulate oxidative stress-induced cell necrosis. H_2_O_2_ is an important factor in the process of oxidative stress. Studies have shown that 500 μM H_2_O_2_ significantly enhances the levels of miRNA-103 and miR-107 in H9C2 cells, and the increase in microRNA-103/107 leads to decreased Fas-associating protein with a novel death domain expression and induces necrotic apoptosis of cardiomyocytes.

“CaMKII is a RIP3 substrate mediating ischemia-and oxidative stress-induced myocardial necroptosis”^[23]^ is another published study on the molecular mechanism of ischemic and oxidative stress-induced necroptosis. The role of PIP3 in myocardial I/R injury was studied by using receptor interaction protein kinase 3 (RIPK3)-deficient mice. RIPK3 deficiency has a protective effect on I/R-induced injured cells. The potential mechanism through which this occurs may involve I/R stimulation of RIPK3 overexpression, upregulation of Ca (2+)-calmodulin-dependent protein kinase (CaMKII) protein levels and regulation of the downstream mitochondrial permeability transition pore leading to the induction of necrotic apoptosis in cardiomyocytes.

### 3.5. Research hotspots and frontier analysis

As shown in Table 5, except for “cardiomyocyte apoptosis” and “apoptosis.” The keywords with high frequency of use were “oxidative stress, expression, activation, heart-failure, inhibition, autophagy, inflammation, mechanisms, I/R injury, protects, and dysfunction,” which appeared more than 500 times, indicating that they represent the main content of research in this field.

**Table 5:** Top 20 keywords in cardiomyocyte apoptosis.

Rank	Keywords	Occurrences	Total link strength	Rank	Keywords	Occurrences	Total link strength
1	apoptosis	2758	14,786	11	cells	563	3224
2	oxidative stress	1324	7378	12	mechanisms	547	3122
3	expression	1038	5698	13	cardiomyocyte	538	3053
4	heart	993	5728	14	ischemia–reperfusion injury	527	3100
5	activation	988	5798	15	injury	523	2948
6	cardiomyocyte apoptosis	753	3841	16	Protects	519	3291
7	heart-failure	701	3851	17	dysfunction	516	2917
8	inhibition	622	3698	18	cardiomyocytes	496	2835
9	autophagy	598	3600	19	Myocardial-infarction	483	2548
10	inflammation	581	3226	20	cell-death	450	2639

**Figure 6.**
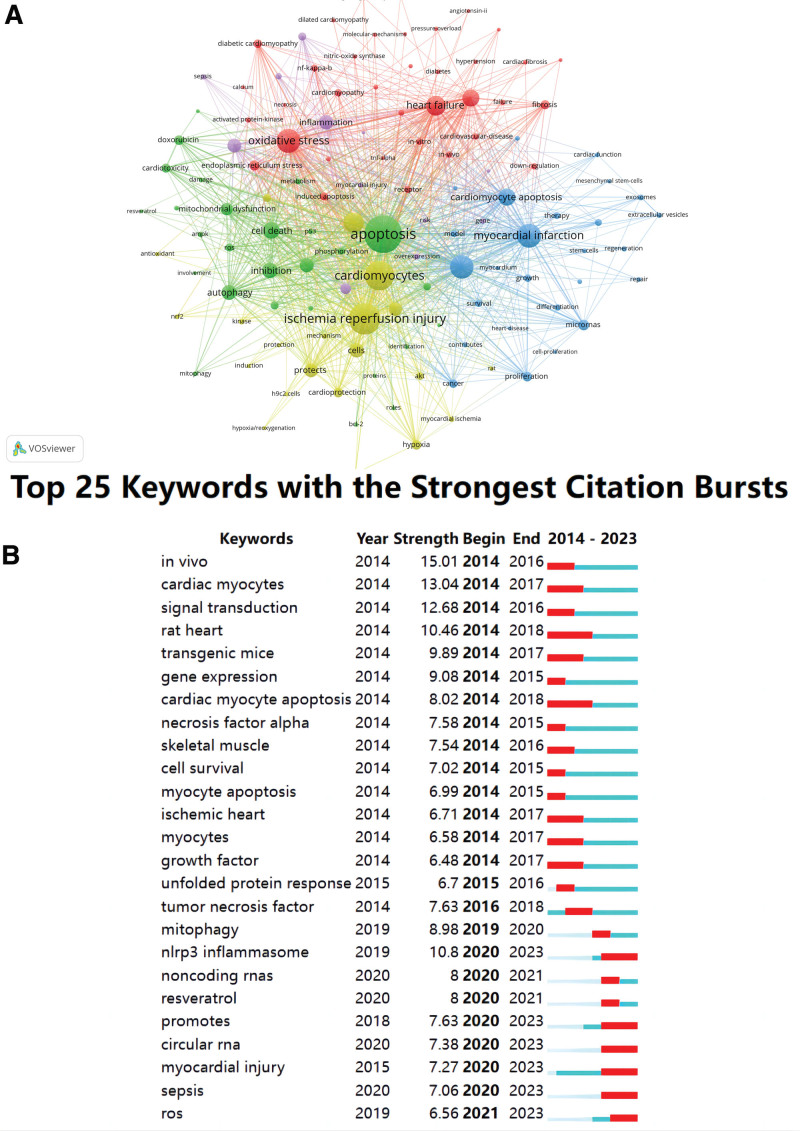
(A) The cluster of keywords in the studies of cardiomyocyte apoptosis. (B) Top 25 keywords with the strongest citation bursts.

Using VOSviewer software parameter settings set to “the minimum frequency of keywords” 80,14,935 keywords were assessed and 122 keywords meet the threshold. The total strength of the 122 keywords and other keywords was calculated, and the keywords with the highest co-occurrence were graphed in Figure 6A. The stronger the connection between nodes, the thicker the curve, and the higher the frequency of the 2 keywords. It can be seen from the figure that the keywords form a total of 5 clusters as follows:

The red cluster mainly contains oxidative stress, heart failure, cardiac hypertrophy, induced apoptosis, necrosis, cardiac fibrosis, endoplasmic reticulum, cardiomyopathy, diabetic cardiomyopathy, activated protein-kinase, nuclear factor-κB, tumor necrosis factor-α, Ca2+, nitric oxide synthase and so on.

The blue cluster mainly contains gene expression, myocardial infarction, survival, heart disease, cardiac function, repair, regeneration, cardiomyocyte proliferation, mesenchymal stem cells, extracellular vesicles, microRNAs, angiogenesis and so on.

The green cluster mainly contains apoptosis, cell death, doxorubicin, autophagy, mitochondrial dysfunction, induced cardiotoxicity, the silent information regulator sirtuin 1, protein kinases B, AMPK, reactive oxygen species (ROS), P53, B-cell lymphoma 2, target, phosphorylation, resveratrol, identification, metabolism and so on.

The purple cluster mainly contains inflammation, cardiac dysfunction, sepsis, myocardial injury, disease, overexpression and so on.

The yellow cluster mainly contains I/R injury, hypoxia/reoxygenation, antioxidant, cardiomyocytes, activation, signaling pathway, cardioprotection, H9C2 cells and so on.

Based on the keyword co-occurrence network, the top 25 keywords with the strongest citation bursts were generated. As shown in Figure 6B. It can be noted that the word “in vivo” (15.01) is the most commonly cited. Followed by cardiac transduction (13.04), signal transduction (12.68), rat heart (10.46), NOD-like receptor thermal protein domain associated protein 3 (NLRP3) inflammasome (10.8), transgenic mice (9.89), gene expression (9.08), mitophagy (8.98), and cardiac myocyte apoptosis (8.02). It can be seen that most of the keywords appeared earlier. NLRP3 inflammasome, promoter, circular RNA, myocardial injury, sepsis, and ROS represent the frontiers of cardiomyocyte apoptosis research and are in their outbreak period.

## 4. Discussion

### 4.1. General information

This paper studies the literature on cardiomyocyte apoptosis published over the past 10 years and uses CiteSpace and VOSviewer software to quantitatively analyze the number of publications, research countries, authors, institutions, literature cocitations and keywords. Finally, the research results and progress are summarized. According to the results, the number of documents published in 2014 was 425, and the overall trend of publications increased over the following 10 years. In 2020 and 2021, the number of publications exceeded 800. The number of citations reflects the research interest in the field. It is obvious from Figure 2 that the number of citations peaked in 2020 and 2021, and was approximately 35,000 in both years. Statistical analysis of the number of documents published by research countries/regions and institutions revealed those with important influence and uncovered their cooperative relations. China, the United States, Canada, Germany and India are the main research countries, and the research from Italy, the United States and Germany is relatively mature. According to the number of publications, the top 10 institutions are all from China. Wuhan University is the institution with the largest number of publications. According to the average citation ranking, institutions within the United States account for three of the top 10 highest citing institutions, and the University of California, San Diego has the highest average citation number. The close cooperation between countries and institutions is conducive to eliminating academic barriers and promoting the development of both academic exchange and cardiomyocyte apoptosis-related research.

Analysis of authors revealed Professors Huang and Chih-yang as the most prolific authors. These authors believe that there are sex differences in cardiomyocyte apoptosis, with more apoptosis observed in males than in females. This increase may be reduced with a decline in estrogen and estrogen receptor function.^[24]^ Many animal experiments have been used to study the molecular mechanism of cardiomyocyte apoptosis induced by fatty acids,^[25]^ hypoxia,^[26]^ high glucose,^[27]^ Adriamycin,^[28]^ and other factors. Adriamycin-induced cardiomyocyte apoptosis is related to ROS-induced damage to mitochondrial function. Heat shock transcription factor 1 and Diallyl trisulfide may be used as potential targets to protect cardiomyocytes.^[29,30]^ Professor Wang, Kun is the author with the highest average citation. He believes that noncoding RNA plays an important role in regulating the biological processes of myocardial cells^[31–33]^ and provides a detailed review of ferroptosis^[34]^ and pyroptosis.^[35]^ The h-index is used to evaluate the comprehensive quantitative index of the number and level of academic output of researchers. Professors Li and Li are the authors with the highest h-index (h-index: 100). In 2015, they published 2 articles establishing the importance of the Hippo pathway in cardiomyocyte regeneration.^[36,37]^ Endoplasmic reticulum stress is another mechanism of through which endogenous apoptosis can be induced.^[38]^ Additionally, he analyzed the molecular mechanism of cardiomyocyte apoptosis induced by sepsis^[39,40]^ and homocysteine.^[41]^

Cardiomyocyte apoptosis occurs in most cardiovascular diseases. Most of the top 10 studies identified in this analysis are related to the molecular mechanism of cardiomyocyte apoptosis. After 2000, it was generally believed that apoptosis was not the only form of programmed cell death. Most forms of cell death, such as autophagy-dependent cell death, ferroptosis, and pyroptosis, are also tightly regulated and are generally referred to as necroptosis.^[42,43]^ Different cell death mechanisms involve different signaling pathways and targets, and accordingly, the treatment of myocardial cell protection will also be diversified. The higher the burst signal in the reference, the higher the intensity of interest in the related research direction and the longer the duration of interest.^[44]^ In the top 20 references, the study of Adriamycin-induced cardiomyocyte apoptosis and its corresponding cytoprotection has attracted considerable attention.^[45–49]^

### 4.2. Hotspots and frontiers

Keyword analysis provides a high-level summary and refinement of the article contents. Through keyword cluster co-occurrence analysis, the research hotspots in this field were determined. Using this approach, keywords were divided into 5 clusters, representing different research topics. The top 25 burst words were analyzed, and the research frontiers and developing trends were determined. The specific contents are as follows:

#### 4.2.1. Cardiomyocyte apoptosis and heart failure.

Red clusters are mainly related to the pathogenesis of heart failure. Exposure of the heart to adverse risk factors, such as pressure overload, volume overload, insufficient blood supply, and high glucose,^[50]^ for a long time leads to pathological myocardial hypertrophy,^[51]^ fibrosis,^[52]^ and other adverse cardiac remodeling. Without timely intervention, heart failure is the ultimate outcome. Cardiomyocyte apoptosis occurs throughout heart failure and is one of the important causes of heart failure occurrence and development. There are 2 main pathways of cardiomyocyte apoptosis: the endogenous pathway (including mitochondria-related and endoplasmic reticulum-related pathways) and the exogenous pathway. The exogenous pathway involves a series of signal transduction pathways that are activated by death ligands (such as Fas and tumor necrosis factor-α). The endogenous pathway involves a variety of signal factors activated by oxidative stress, calcium overload, protein folding errors, etc., leading to the downregulation of antiapoptotic proteins (including B-cell lymphoma 2), activation of effector caspases 3 and 9, apoptotic body production, and ultimately causing cell death.^[53]^ Hsieh et al^[54]^ found that simvastatin can significantly reduce the production of ROS, reduce mitochondrial damage, and inhibit cardiomyocyte apoptosis by inhibiting AngII-mediated cytochrome C and caspase-3 activation. Guo et al^[55]^ found that hyperoside can change the level of apoptosis-related proteins, inhibit apoptosis, and improve cardiac function. Therefore, the study of the activation or inhibition of key apoptotic pathway targets is a hot topic in this field.

#### 4.2.2. Cardiomyocyte apoptosis and cardiac regeneration.

The blue cluster is related to heart regeneration. The regeneration ability of the heart is minimal, and any loss of myocardial cells is basically nonrenewable. Therefore, the key to heart repair is to inhibit of myocardial cell apoptosis and to promote myocardial cell generation. The key targets of intervening in the cardiomyocyte apoptosis pathway, such as fibroblast growth factor 21^[56]^ and adenosine monophosphate-activated protein kinase,^[57]^ can reduce cardiomyocyte apoptosis. In addition, there are 3 ways to supplement the loss of cardiomyocytes. The first is to stimulate existing mature cardiomyocytes to reenter the cell division cycle; for example, Volland et al^[58]^ showed that breast cancer susceptibility gene-associated protein can downregulate the expression of p21Cip, regulate the cell cycle, and promote cardiomyocyte proliferation. Second, gene therapy can be used to convert noncardiomyocytes into cardiomyocytes.^[59]^ The third is stem cell transplantation. Studies have shown that the microenvironment of myocardial infarction can activate monocytes in bone marrow and promote their secretion of exosomes rich in miRNA. Exosomes migrate to the injured site to play a role in cardiac repair. For example, hypoxic preconditioning bone marrow mesenchymal stem cell-derived exosomes containing miR-19a^[60]^ and miR-22^[61]^ are transferred to damaged cardiomyocytes, which plays an important role in exosome-mediated anti-apoptosis. However, pluripotent stem cell-derived cardiomyocytes can lead to ventricular arrhythmia. However, allogeneic stem cell transplantation has the risk of immune rejection. The method of promoting direct reprogramming of nonmyocardial cells into cardiomyocytes is inefficient and has long-term safety problems. Therefore, evaluating the risk-benefit ratio of each therapy and approaches to reconstruct damaged cells and blood vessels remain hot research topics in cardiac regeneration.

#### 4.2.3. Cardiomyocyte apoptosis and Adriamycin-induced cardiomyopathy.

The green cluster is mainly related to Adriamycin-induced cardiomyopathy. Adriamycin is a highly effective anthracycline chemotherapeutic drug for the treatment of tumors, but it can be dose-dependently toxic to the heart.^[62]^ Cardiomyocyte apoptosis and oxidative stress are currently believed to be the main causes of doxorubicin-induced cardiotoxicity.^[63]^ Doxorubicin can initiate the adenosine monophosphate-activated protein kinase/mammalian target of rapamycin autophagy-related pathway and prevent the degradation of lysosomes, leading to the accumulation of autophagosomes and lysosomes in cells and promoting the production of ROS and cell death.^[64]^ Second, doxorubicin can increase intracellular free iron,^[65]^ activate the NF-E2-related factor 2/heme oxygenase 1 pathway, and induce cardiomyocyte ferroptosis,^[66]^ and the RIPK3/CaMKII/mitochondrial permeability transition pore signaling pathway can also lead to programmed cell necrosis.^[67]^ Following NLRP3 upregulation, Gasdermin D is cleaved, the cell membrane is perforated, cell pyroptosis is induced,^[68]^ P53 is upregulated, and apoptosis is induced by endogenous and exogenous pathways^[69]^ leading to cardiomyocyte damage. Studies have shown that overexpression of SIRT1, 3, and 6 can attenuate doxorubicin-induced apoptosis and mitochondrial oxidative stress damage.^[70–72]^ In addition, traditional Chinese medicine extracts such as sesamin and resveratrol^[73]^ not only have anti-inflammatory and antioxidant activities but can also maintain mitochondrial function stability by activating the expression of the endogenous apoptosis inhibitor SIRT-1 in mitochondria and upregulating the level of manganese superoxide dismutase, thereby exerting anti-Adriamycin cardiotoxicity. However, there is still a lack of effective measures to prevent or treat doxorubicin cardiotoxicity in clinical practice. Therefore, exploring the mechanism of doxorubicin cardiotoxicity and identifying effective defense targets are important research directions to improve the survival rate of cancer patients.

#### 4.2.4. Cardiomyocyte apoptosis and I/R.

The yellow cluster is mainly related to I/R injury. In the event of acute palpitation infarction, due to the interruption of blood supply, some myocardial cells undergo ischemic necrosis, and the death of some cells is caused by reperfusion injury.^[74]^ Studies have shown that in the early stage of myocardial infarction, even over a few months,^[75]^ apoptotic cells in the infarcted area can be observed. Because the regeneration ability of myocardial cells is very limited, understanding how to prevent reperfusion injury and reverse apoptosis^[76]^ requires continued exploration by researchers.

#### 4.2.5. NLRP3 inflammasome, circular RNA, and sepsis are the frontiers of research in this field and are currently in an explosive phase.

##### 4.2.5.1. NLRP3 inflammasome.

NLRP3 is the first research frontier identified in this study. NLRP3 is widely expressed in immune cells such as monocytes-macrophages, dendritic cells, and neutrophils. As a cytoplasmic multiprotein complex, NLRP3 is extremely sensitive to danger signals and can be used to identify them. Pathogen-associated molecular patterns and damage-associated molecular patterns are involved in the body’s inflammatory response and their excessive activation is involved in a variety of cell death pathways,^[77]^ leading to long-term chronic inflammation. At present, there are 4 recognized mechanisms of NLPR inflammasome activation in the heart: ion flow, ROS, endoplasmic reticulum stress and lysosomal rupture or cathepsin B release.^[78]^ In addition, the of Golgi activation of NLRP3 has gradually become a concern. For example, NLRP3 forms a trimer with SREBP cleavage-activating protein and sterol regulatory element-binding protein 2, cotranslocates to the Golgi, and participates in the transcription of genes related to cholesterol synthesis and metabolism and the assembly and activation of inflammasomes.^[79]^ The elucidation of the role of the Golgi in activating inflammatory pathways is currently in the early stages of research, so research focusing on the specific mechanisms in this field will become a hotspot.

In addition, there is evidence that aseptic inflammation caused by myocardial I/R injury is mediated by the NLRP3 inflammasome,^[80]^ and the expression of NLRP3, interleukin-1 and interleukin-18 mRNA in noncardiomyocytes is significantly higher than that in cardiomyocytes.^[81]^ In pressure overload-induced heart failure, CaMKIIδ is involved in NLRP3-mediated inflammation, leading to myocardial hypertrophy and fibrosis.^[82–84]^ Han et al^[83]^ have shown that after the deacetylase SIREI is activated by agonists, it inhibits ROS-mediated NLRP3 inflammasome activation through the protein kinases B/cardiac pyruvate dehydrogenase signaling pathway and improves myocardial cell pyroptosis caused by I/S injury. Knockout of NLRP3 can reduce myocardial fibrosis.^[84]^ Therefore, inhibiting the expression of NLRP3 and improving the effectiveness and safety of treatment are likely to be the focus of future research.

##### 4.2.5.2. Circular RNA.

CircRNA is involved in the regulation of autophagy and apoptosis in cardiomyocytes. For example, the expression of circHIPK3 is upregulated during myocardial I/R injury. It can not only improve the autophagy level of cardiomyocytes through the circHIPK3/miR-20b/autophagy-related protein 7 signaling pathway^[85]^ but also activate the apoptosis program,^[86]^ aggravating I/R injury. circRNAs have the characteristics of extensiveness, conservation and tissue specificity, and gene regulatory pathways composed of various circRNAs, mRNA and miRNAs are interconnected to form a rich network. With the continuous development of molecular biotechnology, it is increasingly possible to carry out more involved epigenetic research and then modify and regulate the target gene by using circRNA. However, at present, circRNA research is still in its infancy, and the discovery of new circRNAs or the exploration of their mechanism of action still has a long way to go.

##### 4.2.5.3. Sepsis.

Sepsis is a common disease in the intensive care unit with high morbidity and mortality. When sepsis occurs in the body due to infection, trauma and other factors, it can directly activate the body’s innate immune response, which in turn causes inflammation and the release of myocardial inhibitory factors, resulting in oxidative stress^[87,88]^ and the gradual development of cytokine storms,^[89]^ mitochondrial dysfunction^[90,91]^ and abnormal energy metabolism,^[92]^ ultimately affecting myocardial function, in a diseases known as septic cardiomyopathy (SCM). The main features of SCM are no coronary occlusion and reversibility.^[93]^ In SCM disorder is more obvious at the subcellular level than at the tissue structure level, indicating that the cardiac dysfunction of SCM is a functional disorder rather than a structural disorder.^[94,95]^ If the risk period can be passed, the cardiac dysfunction caused by SCM is reversed and cardiac function will return to normal in 7 to 10 days. Clinical treatment of SCM mainly includes intervening in septic shock, but a large amount of fluid resuscitation aggravates cardiac load. Studies have shown that autophagy^[96,97]^ and noncoding RNA^[98–100]^ are important in the pathogenesis of SCM, and interventions targeting autophagy and noncoding RNA have become a new direction for the treatment or study of SCM.

## 5. Conclusion

In this study, VOSviewer and Citespace were used to analyze the literature related to cardiomyocyte apoptosis to intuitively reveal the research status and frontier trends in this field. Since the literature retrieved in this study is limited to the Web of Science core database, the results may have certain limitations. The research hotspots of cardiomyocyte apoptosis are constantly changing over time. The research hotspots mainly focus on experimental research, molecular mechanisms, pathophysiology and cardiac regeneration of related diseases. The NLRP3 inflammasome, circular RNA, and sepsis were identified as research hotspots in this field. The apoptosis pathways activated by different induction conditions differ. The in-depth study of the network signaling pathways of apoptosis, identifying important intervention targets, reducing apoptosis, and improving cardiac function still requires continuous research efforts.

## Acknowledgments

Citespace. was invented by Professor CM Chen and VOSviewer. was invented by Leiden University in the Netherlands, both of which are free to use. The author expresses gratitude and appreciation for this.

## Author contributions

**Data curation:** Rui Wang, Xu Luo, Songyun Li, Xin Wen, Xin Zhang, Yunxiang Zhou.

**Methodology:** Wen Xie.

**Writing – original draft:** Rui Wang, Xu Luo.
